# Liquid-curtain-based strategy to restrain plume during flushing

**DOI:** 10.1063/5.0033836

**Published:** 2020-11-01

**Authors:** Su-Chen Wu, Meng-Yue Guo, Ji-Xiang Wang, Shuhuai Yao, Jun Chen, Yun-yun Li

**Affiliations:** 1 College of Electrical, Energy and Power Engineering, Yangzhou University, Yangzhou 225009, People’s Republic of China; 2 Key Laboratory of Energy Thermal Conversion and Control of Ministry of Education, School of Energy and Environment, Southeast University, Nanjing 210096, People’s Republic of China; 3 Department of Mechanical and Aerospace Engineering, The Hong Kong University of Science and Technology, Kowloon, Hong Kong, People’s Republic of China

## Abstract

How to prevent the flushing-induced plume without changing people’s daily habits? Enlightened by thoughts of redesigning the restroom, this article provides a redesigned toilet using liquid-curtain-based strategy and verifies its advantages from the computational fluid dynamics. Two favorable effects are spotted: (1) the liquid curtain can suppress the upward virus particles (only 1% viruses can be lifted out of the toilet) and (2) the flow distribution caused by the liquid curtain can deliver virus particles into the sewage efficiently.

In December 2019, COVID-19 pandemic first broke out in Wuhan, China. COVID-19 is caused by a highly infectious novel coronavirus “SARS-CoV-2.”^1^ It spread from Wuhan to the whole country in just one month^1^ and then to the whole world in a few months.^2,3^ At present, millions of people have been reported to be infected by “SARS-CoV-2” across the world,^4^ causing panic and economic loss all over the world. Airborne transmission has been confirmed to be the main channel of infection.^5,6^ In addition, several studies have shown that fecal-oral transmission could be one of the transmission channels.^7,8^ Scientists even detected “SARS-CoV-2” from urine of a COVID-19 patient.^9^ All the evidence shows not only public restrooms but family bathrooms could become “dangerous” places of possible virus sources. Our previous study has demonstrated that flushing processes of both toilets and male urinals could cause a massive spread of the virus.^10,11^ Global Times has confirmed that two of Beijing’s COVID-19 cases were infected from a contaminated public restroom.^12^ Besides COVID-19 patients, patients of Ebola, SARS, Hepatitis A, etc., can also cause cross-infection when using the toilet.^13,14^

Putting the toilet lid down before flushing can be an effective method to prevent the plume caused by the flushing of a toilet. However, a male urinal does not have a lid or other barriers which are able to prevent the virus transmission, and people do not always have the habit of putting the toilet lid down before flushing. Therefore, “how to prevent flushing-induced virus transmission without changing people’s daily habits?” is an urgent public concern that needs to be responded to. Enlightened by the report regarding rethinking the design of restrooms,^15^ the authors adopt a liquid-curtain-based strategy as a protective barrier to eliminate the plume. The focus of this letter is a novel siphon toilet in which the liquid-curtain is ejected to prevent the toilet plume. Results from computational fluid dynamics (CFD) can illustrate the advantage of the application of the proposed liquid-curtain approach quantitatively.

Figure 1(a) shows the 3D model of the proposed toilet with a liquid-curtain which is generated by the liquid feed module (LFM) and the semi-circular pain nozzle (inlet 2). A pump inside the LFM can control the “on” an “off” of the liquid-curtain. Detailed size information of the focused model and the initial phase distribution inside it are shown in Fig. 1(b). Inlet 2 is located at the left side of the bowl, 0.03 m below the toilet seat. The blue block shown in Fig. 1(b) represents the air area, and the pink represents the water. Figure 2 demonstrates the meshing of the model. The total number of the grids is 14 756, which is determined by sensitivity analysis. Figure 3 shows the initial distribution of assumed virus-laden particles, the total number of which is 6300. The flushing process with the ejected liquid-curtain is modeled by volume of fluids (VOF), which specializes in tracking and characterizing the liquid–gas two-phase interface.^16^ In addition, the movement of particles is analyzed by the discrete phase model (DPM), which has successfully predicted the cough-induced aerosol movement^17,18^ and sprayed droplet flow^19–21^ and other particle-like movement. The boundary condition of inlet 2 is set as the velocity-inlet with a velocity magnitude of 2.6 m/s (see the supplementary material for the reason to choose this velocity). For detailed model establishment, simulation assumption, and boundary conditions, refer to our previous work.^10,11^

**FIG. 1.**
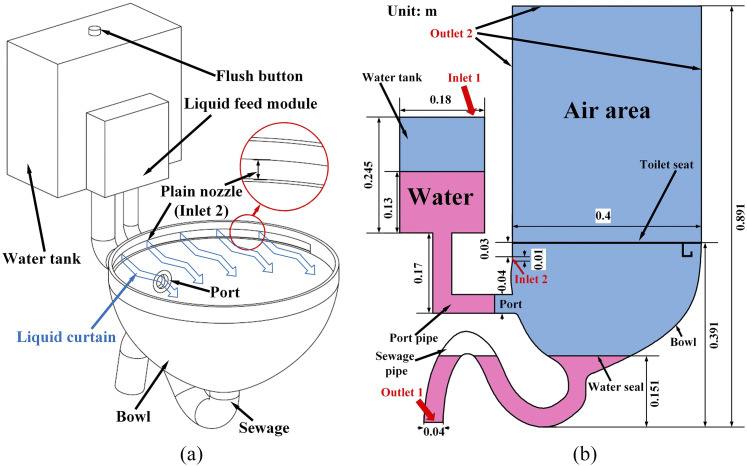
Schematic of the (a) redesigned toilet and (b) focused physical model.

**FIG. 2.**
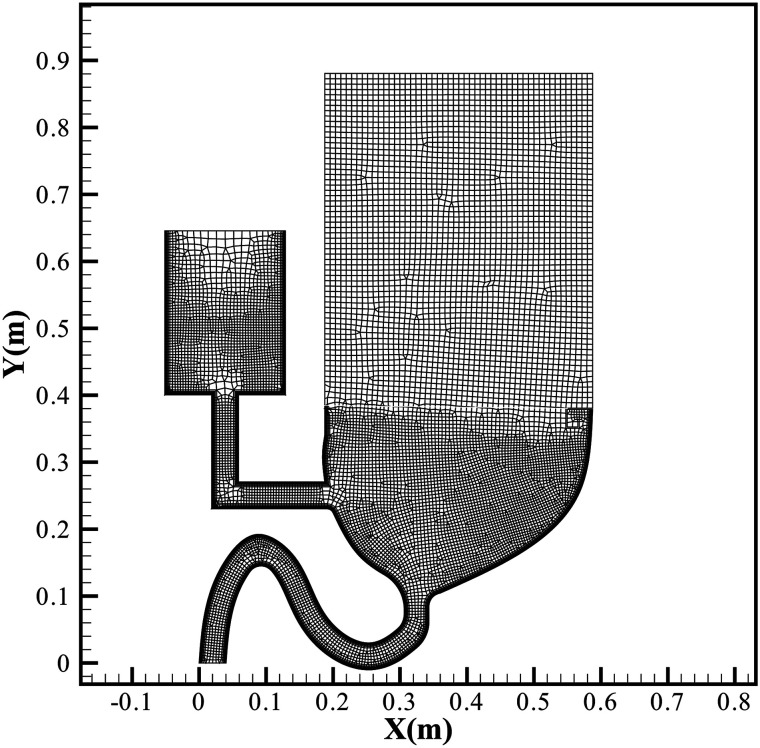
Meshing.

**FIG. 3.**
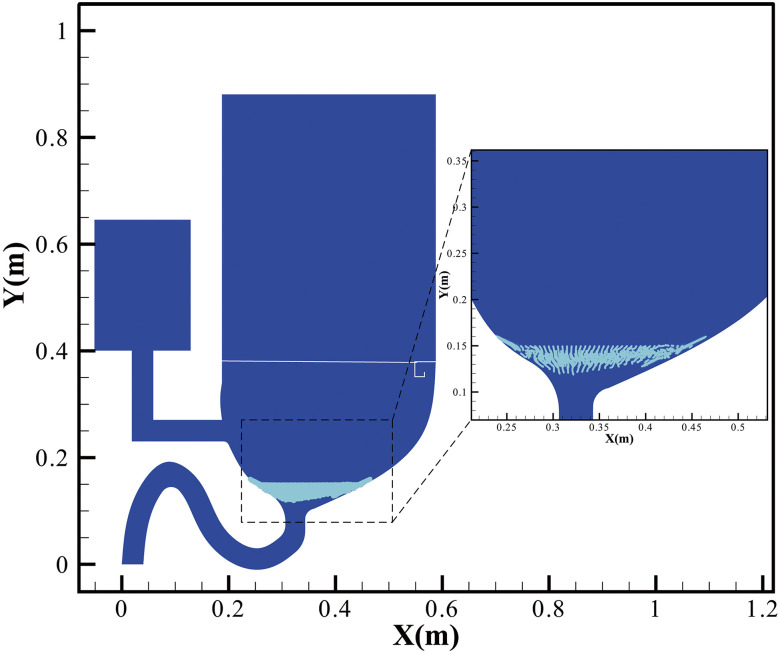
Initial distribution of virus-laden particles.

Figure 4 demonstrates the movement of virus-laden particles with the liquid-curtain (multimedia view) where the majority of the particles are prevented from spreading out. It can be seen that most particles are suppressed by the liquid current and travels with the liquid-curtain flow from the left to the right bowl wall. Additionally, comparison of the particle-involved flushing processes with and without the liquid-curtain is shown in Fig. 5, where particle distribution at 12.1 s is presented. Figure 5(a) shows that a large portion of the virus-laden particle is lifted up to the air, which is coherent with Li’s work where 45%–60% virus particles will be brought out.^10^ In contrary, most of the virus-laden particles are washed away with the assistance of the liquid-curtain, as shown in Fig. 5(b). Data statistics show that about only 1% of virus-laden aerosols enter the air area above the toilet seat in the latter case.

**FIG. 4.**
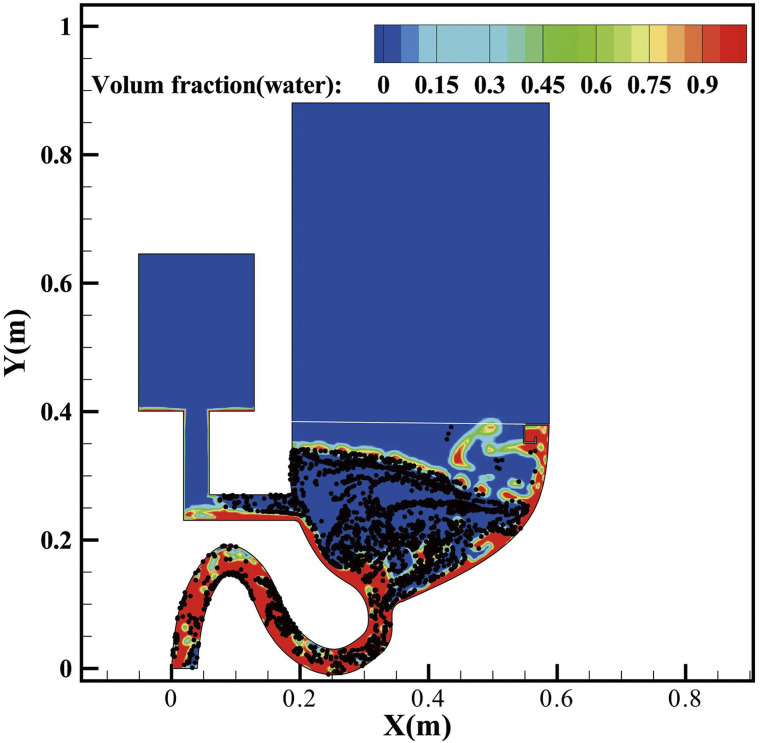
Dynamic movement of particles during toilet flushing with the liquid-curtain barrier. Multimedia view: https://doi.org/10.1063/5.0033836.110.1063/5.0033836.1

**FIG. 5.**
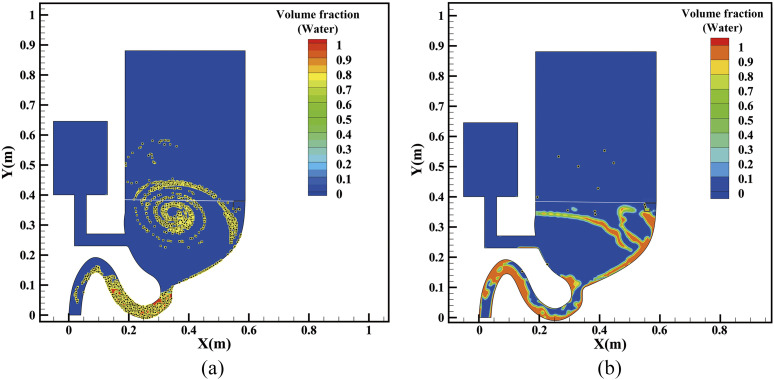
Comparison of virus movements (a) without the liquid curtain and (b) with the curtain.

Figure 6 presents the velocity magnitude and particle distributions at different times. It can be seen in Fig. 6(a) that the particle distribution is coherent with the velocity distribution where the particles are always delivered to the dark-color area which represents the relatively high velocity area because the particle mass is so small that it can easily be affected by the liquid-curtain flow with strong kinetic energy. Combined with Fig. 7, which illustrates the velocity vector distribution, it can be explained that the liquid-curtain impacting effect creates a downward flow distribution that can bring the trapped particles down into the sewage pipe. Figures 6(b) and 6(c) illustrate the particle leakage location which has been marked with red circles under the protection of the liquid-curtain. It shows that most leakage points occur in the light area where the velocity magnitude is relatively low as the liquid-curtain is hindered by the bowl wall and decelerated. The water in that area will be splashed into discrete droplets, so the particle can be charged out from gaps between these discrete droplets. Refer to the supplementary material for the time-varying distribution of pressure for another explanation of restraining the plume.

**FIG. 6.**
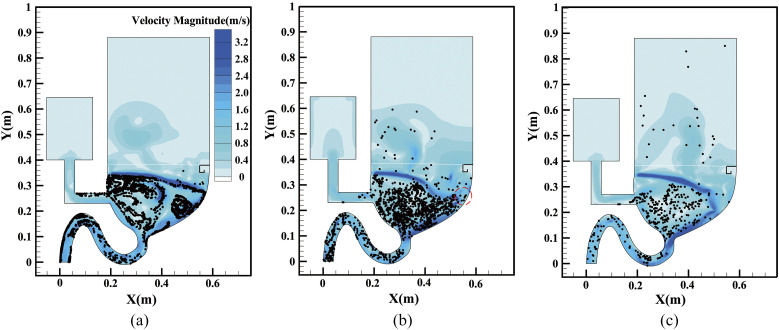
Velocity and particle distribution at different times: (a) 0.9 s, (b) 2.1 s, and (c) 4.6 s.

**FIG. 7.**
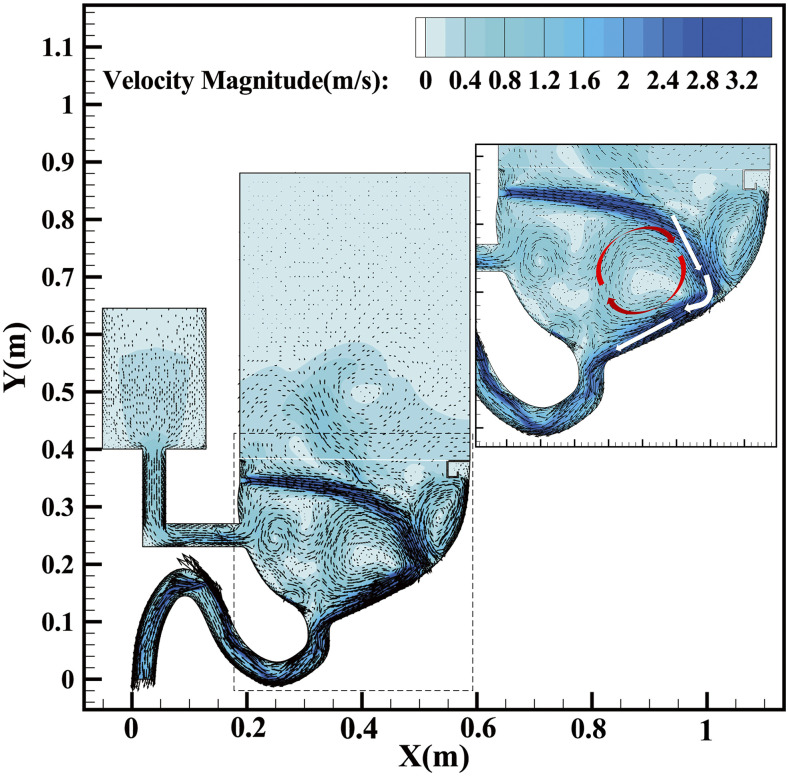
Plots of velocity magnitude and vector at 1.885 s.

In conclusion, a redesigned toilet equipped with a liquid-curtain can effectively impede upward movement of the virus particle. Two favorable effects are identified: (1) the ejected horizontal liquid-curtain can suppress the upward movement of the particles; (2) the downward velocity distribution caused by the curtain impact can deliver these particles into the sewage pipe and wash them away. Numerical data show only 1% particles can be lifted out of the redesigned toilet. In addition, such a liquid-curtain-based strategy can also be applied to male urinals to prevent virus transmission.

See the supplementary material for reasons to select the velocity of inlet 2 (2.6 m/s) and time-varying distribution of pressure during flushing with the liquid curtain.

## DATA AVAILABILITY

The data that support the findings of this study are available from the corresponding author upon reasonable request.
